# Determinants of antigenicity and specificity in immune response for protein sequences

**DOI:** 10.1186/1471-2105-12-251

**Published:** 2011-06-21

**Authors:** Yulong Wang, Wenjun Wu, Nicolas N Negre, Kevin P White, Cheng Li, Parantu K Shah

**Affiliations:** 1 Department of Biostatistics and Computational Biology, Dana-Farber Cancer Institute & Harvard School of Public Health, Boston 02115 MA, USA; 2 State Key Laboratory of Software Development Environment, Beihang University, Beijing, 100191, China; 3 Institute for Genomics and Systems Biology, The University of Chicago, Chicago 60637 IL, USA

## Abstract

**Background:**

Target specific antibodies are pivotal for the design of vaccines, immunodiagnostic tests, studies on proteomics for cancer biomarker discovery, identification of protein-DNA and other interactions, and small and large biochemical assays. Therefore, it is important to understand the properties of protein sequences that are important for antigenicity and to identify small peptide epitopes and large regions in the linear sequence of the proteins whose utilization result in specific antibodies.

**Results:**

Our analysis using protein properties suggested that sequence composition combined with evolutionary information and predicted secondary structure, as well as solvent accessibility is sufficient to predict successful peptide epitopes. The antigenicity and the specificity in immune response were also found to depend on the epitope length. We trained the B-Cell Epitope Oracle (BEOracle), a support vector machine (SVM) classifier, for the identification of continuous B-Cell epitopes with these protein properties as learning features. The BEOracle achieved an F1-measure of 81.37% on a large validation set. The BEOracle classifier outperformed the classical methods based on propensity and sophisticated methods like BCPred and Bepipred for B-Cell epitope prediction. The BEOracle classifier also identified peptides for the ChIP-grade antibodies from the modENCODE/ENCODE projects with 96.88% accuracy. High BEOracle score for peptides showed some correlation with the antibody intensity on Immunofluorescence studies done on *fly *embryos. Finally, a second SVM classifier, the B-Cell Region Oracle (BROracle) was trained with the BEOracle scores as features to predict the performance of antibodies generated with large protein regions with high accuracy. The BROracle classifier achieved accuracies of 75.26-63.88% on a validation set with immunofluorescence, immunohistochemistry, protein arrays and western blot results from Protein Atlas database.

**Conclusions:**

Together our results suggest that antigenicity is a local property of the protein sequences and that protein sequence properties of composition, secondary structure, solvent accessibility and evolutionary conservation are the determinants of antigenicity and specificity in immune response. Moreover, specificity in immune response could also be accurately predicted for large protein regions without the knowledge of the protein tertiary structure or the presence of discontinuous epitopes. The dataset prepared in this work and the classifier models are available for download at https://sites.google.com/site/oracleclassifiers/.

## Background

The humoral immune response is based on the ability of antibodies to recognize and bind to epitopes on the surface of antigens with high specificity. It is believed that most protein epitopes are composed of different parts of the polypeptide chain that are brought into spatial proximity by the folding of the protein or discontinuous. However, for approximately 10% of the epitopes, the corresponding antibodies are cross-reactive with a linear peptide fragment of the epitope [1]. These epitopes are termed linear or continuous and are composed of a single stretch of the polypeptide chain.

In many cases it is difficult to obtain a pure preparation of the protein of interest for immunization purposes. The traditional cloning of the proteins or experimental peptide scanning approach is clearly not feasible on a genomic scale. However, to raise antibodies it is not necessary to present the complete protein but only the immunogenic fractions. Specific antibodies can be generated by immunization of animals with a peptide if the peptide is well chosen and presents an effective continuous epitope of the protein. The continuous B-cell epitopes play a vital role in the development of peptide vaccines, in diagnosis of diseases, and for allergy research. The specific interactions between antibodies generated against the continuous epitopes are also exploited extensively in biochemical and high-throughput assays. The ENCODE [2] and the modENCODE [3] projects aim to profile protein-DNA interactions for all transcription factors and DNA associated proteins for Human and for model organisms like *Drosophila melanogaster *and *Caenorhabditis elegans *using the factor specific antibodies. This has increased the demand for good antibodies at the whole genome level.

The computational methods can be cost effective and reliable for predicting linear B-cell epitopes and can guide a genome wide search for antigenic B-cell epitopes. Therefore, a lot of research has been devoted in the past for identifying continuous B-cell epitopes from the protein sequences. The classical approach of epitope prediction is to utilize the amino acid propensity scales describing properties like hydrophobicity [4], hydrophilicity [5], flexibility/mobility [6], surface accessibility [7], polarity [8,9], turns [10], and antigenicity [11]. The first propensity scale method for predicting linear B-cell epitopes was introduced by Hopp and Woods [12] and utilized the Levitt hydorophilicity scale [13] to assign a propensity value to each amino acid. PREDITOP [10], PEOPLE [14], BEPITOPE [15], and BcePred [16] predicted linear B-cell epitopes based on combinations of physico-chemical properties as opposed to the propensity measures that rely on individual properties. The BcePred method obtained the best specificity of 56% and sensitivity of 61% [16]. Blythe and Flower assessed 484 amino acid propensity scales in combination with ranges of plotting parameters and found that even the best set of scales and parameters perform only marginally better than random [17]. This led researchers to combine propensity scales with machine learning methods to improve the performance. The BepiPred [1] method combined the Parker hydorophilicity scale [5] with a Hidden Markov Model (HMM) and demonstrated a slight but statistically significant improvement in the classification performance compared to the performance of the propensity scale based methods. Chen *et al*. [18] developed an amino acid pair (AAP) antigenicity scale that assigns to each possible pair of amino acids, a propensity value. Their support vector machine (SVM) classifiers trained using amino acid pair (AAP) propensity derived features outperformed SVM classifiers trained using amino acid propensity derived features [18].

Recently, several researchers have explored various machine learning methods with learning examples for predicting linear B-cell epitopes using amino acid sequence information. The ABCPred [19] method use recurrent artificial neural networks for predicting linear B-cell epitopes. Söllner and Mayer [20] represent each peptide features derived from a variety of propensity scales, neighborhood matrices, and respective probability and likelihood values and attained an accuracy of 72%. The BCPred and FBCPred [21] methods predict linear B-cell epitopes and flexible length linear B-cell epitopes (respectively) using SVM classifiers that use string kernels. The COBEpro [22] method use a two-step procedure for predicting linear B-cell epitopes. In the first step, an SVM classifier is used to assign scores to fragments of the query antigen. In the second step, a prediction score is associated with each residue in the query antigen based on the SVM scores for the peptide fragments. Many methods utilizing three-dimensional (3D) structure to predict discontinuous epitopes are also available [23,24]. We refer readers to a recent review by El-Manzalawy and Honavar [25] for a more detailed discussion.

There are several problems common to recently developed machine-learning methods. These methods have utilized only a limited amount of positive learning examples. Some of these methods have utilized negative learning examples derived from random protein fragments. These negative training examples may harbor genuine B-cell epitopes and affect the training procedure and result in poor classification performance. Moreover, none of the published work has systematically combined and compared the performance of various structural properties and evolutionary information in bringing about good classification performance. Finally, most methods have utilized large peptide lengths (e.g. 20) in their benchmarking experiments. Predicting protein epitopes within the length range of 7-15 is important as peptide in this length range are easy to synthesize experimentally and well-chosen peptides could generate specific antibodies. The effect of peptide length on the classification performance has not been checked systematically. It is worth noting that these methods have failed to achieve accuracy > 75% and AUC > 0.75.

To overcome these limitations, we have generated a large non-redundant training set of B-cell epitopes with both the positive and the negative learning examples. This dataset was prepared by combining data from the Immune epitope database [26], the BCIPEP database [27] and the AntiJen database [28,29] that has information on small peptide epitope antigenicity and specificity in immune response. We have created and leveraged the computational capabilities of Open Life Science Gateway (OLSGW; Wu *et al*. Submitted; see Methods) to exhaustively predict structural properties for sequences containing epitopes. We have checked contributions of different protein structural properties including first and higher order composition, evolutionary conservation information, compositional and per residues probabilities for secondary structure, solvent accessibility, disorder, and low-complexity. We show that the utilization of negative examples and increasing the training set size contribute to the improved classification performance. However, the set of learning features have a definite impact on the learning process. We have also checked the effect of epitope length on the classification performance.

We introduce the B-cell epitope oracle (BEOracle), a SVM classifier that integrates different types of information that was provided as learning features to the classifier and validate the classifier on multiple validation sets. A large test set, a list of 32 chromatin immunoprecipitation (ChIP) grade antibodies and 10 HDAC antibodies with known epitope sequences were utilized as validation sets. Moreover, we checked whether BEOracle scores for HDAC peptides antibodies correlated with the intensity of fluorescence in immunofluorescence experiments on the *Drosophila melanogaster *embryos. Finally, a second SVM classifier, B-cell region oracle (BROracle) that utilizes the BEOracle scores as learning features, was trained to predict specificity of antibodies produced after immunization with large protein domains. This is first such attempt in our knowledge. Validation information for immunofluorescence (IF), western blot (WB), immunohistochemistry (IHC) and protein array (PA) data from Protein Atlas database was used to assess performance of BROracle.

## Results

**Figure 1:**
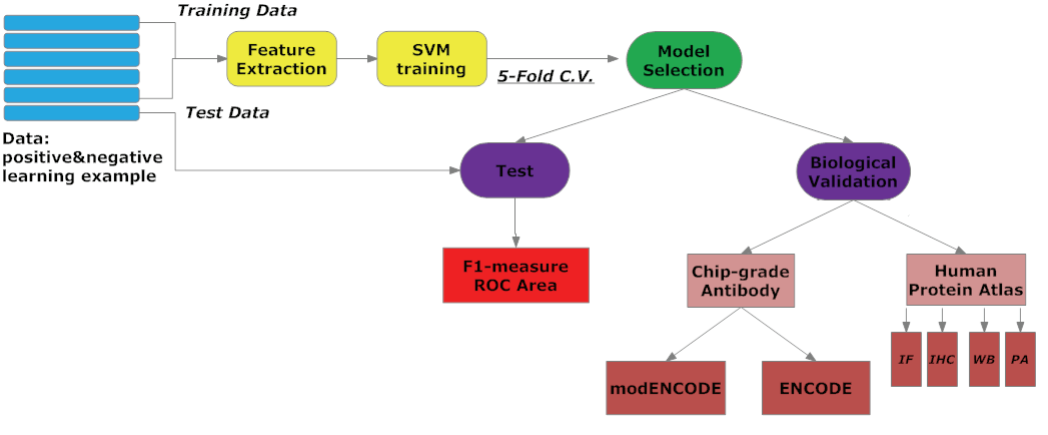
**An overview of the machine learning and validation procedures for BEOracle and BROracle SVM classifiers**.

We have carried out more than 150 SVM classification experiments with a large learning set that provides both positive and negative learning examples for the training of the BEOracle and the BROracle classifiers (Figure 1). Each classification experiment includes a 5-fold cross validation on the training set while varying the featuresets, the SVM kernels and the peptide lengths. The learning features extracted from training examples provided to the classifier protein composition information in terms of the n-grams, evolutionary information encoded in the position specific scoring matrix (PSSM), and the structural information like secondary structure, solvent accessibility, disorder, and low-complexity assignments predicted using the state-of-the-art bioinformatics algorithms. The BEOracle and the BROracle classification performances were validated on multiple validation sets. See Methods for details on learning sets and feature extraction procedure.

### Combining sequence features improve classification performance

**Figure 2:**
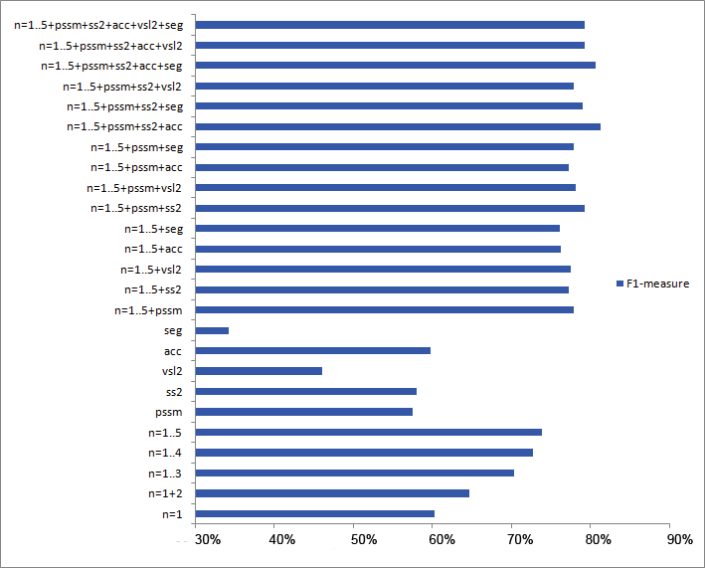
**The SVM classifier performance on single features and on various feature combinations**. N-gram features (n = 1..5) provide compositional information, secondary structure (ss2), solvent accessibility (acc), disorder (vsl2) and low-complexity features (seg) provide structural information, and positional specific similarity matrix (pssm) that provides evolutionary information, are used in different combination to identify a set of features that can provide best classification performance.

Our experiments suggest that the n-gram features providing information on the protein composition with length 15 provides good classification performance and generalizes well (Figure 2). The classification performance calculated as the F1-measure decreases from 73.95% to 60.32% as n-gram number decreases from 5 to 1 (see Methods). Using position based encoding, normalizing the n-grams with background frequency of amino acids in Uniprot database or using physico-chemical scales for SVM learning did not provide good classification performance (Additional file 1 Section 6).

Our results indicate that no structural feature alone provides satisfactory classification performance. On the other hand, combining appropriate features substantively improves the classification accuracy and the F1-measure (Figure 2). The combination of n-gram features, evolutionary information, secondary structure and solvent accessibility information resulted in the best classification performance (*best feature set*; Figure 2). The best 3 classifiers in the 5-fold cross validation studies utilized the *best feature set *with polynomial kernels of degrees 2 and 3, and the radial basis function kernel (RBF), respectively. Adding other structural features such as disorder and low complexity information did not improve classification performance (Figure 2).

**Figure 3:**
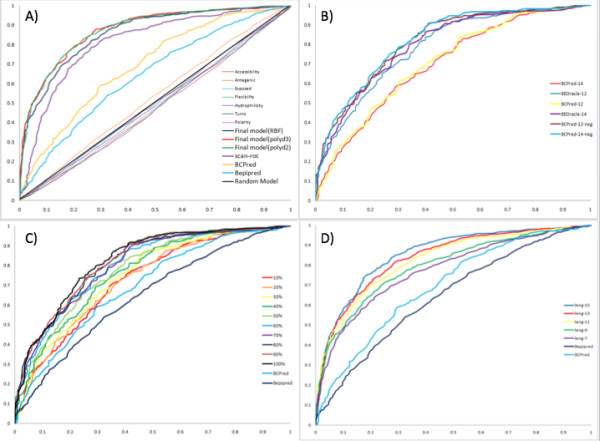
**Comparision of classifier performance:(A)** performance of the best classifier at length 15 compared to other methods, **(B)** impact of negative examples on SVM learning and comparison of the classifier performance with BCPred at lengths 12 and 14, **(C)** Effect the of training dataset size on the classification performance and **(D)** comparision of best classifiers at different epitope lengths.

The best classifier achieved an accuracy of 82.16% and the F1-measure of 81.37% to the average accuracy of 59.51% and the average F1-measure of 59.08% when using only one type of features. The polynomial kernel of degree 3 has the highest area under the curve (AUC) of 0.88 followed by the polynomial kernel of degree 2 with the AUC of 0.87 and the RBF kernel with the AUC of 0.86 (Figure 3A). The BCpred [18] and Bepipred [1] methods follow with AUC = 0.70 and AUC = 0.65, respectively. The identification of epitopes using various amino acid propensity scales is only marginally better than the random model as mentioned above. The classification performance of our SVM classifier(s) is a marked improvement on these and other published methods.

### Utilization of negative training examples improve the classification performance

To assess the contribution of negative learning examples on the SVM classification performance; we trained our best SVM classifier with a dataset prepared by El-Manzalawy *et al *[21] and the *best feature set*. The El-Manzalawy dataset contains 777 non-redundant positive training examples from BCIPEP database [27] and an equal number of negative learning examples randomly extracted from the sequences in the Swissprot database. The SVM classifier trained on the random negative examples achieved the AUC of 0.7813 (F1-measure of 73.90%) at the epitope length of 12 and the AUC of 0.7881 (F1-measure of 74.49%) at the epitope length of 14 (Figure 3B). This is a small yet significant decrease in classification performance compared to the AUC of 0.7951 (F1-measure of 75.92%) and 0.8019 (F1-measure of 76.12%) obtained when the classifier was trained with actual negative examples at these lengths (see Additional File 1, Section 8).

The training of our SVM classifier on this dataset has also allowed for a more direct comparison of the prediction performance of our classifier with the BCPred method (Figure 3B). The BCPred classifier achieved the AUC of 0.7135 and 0.7145 at lengths of 12 and 14 on the test set prepared by us, which is significantly less than the AUC achieved by our classifier mentioned in the previous paragraph. Thus, our classifier even when trained on El-Manzalawy dataset clearly outperforms BCPred (Figure 3B). This result also supports the conclusion that utilization of the best feature set improves the classification performance (see Additional File 1, Section 8).

### Improvement in the classification performance is not solely dependent on the size of the training set

Compared to other published methods we have created a large learning set that provides the SVM classifier with both the positive and the negative learning examples. It is possible that this large learning set size is solely responsible for the improved classification performance. Therefore, we utilized 10% (392 positive and 392 negative examples) increments of our training set with epitope length 15 and the *best feature set *to study the performance improvement with increase in the learning set (Figure 3C). The F1-measure decreases from 81.37% to 71.31% with the decrease in learning examples and the AUC decreases from 0.88% to 0.75%. However, even when utilizing just 10% of learning examples, which is less than the training set size of BCPred or Bepipred, the SVM classifier trained on the *best feature set *outperforms these methods.

### Classification performance is dependent on the epitope length

The epitope length of 15 utilized in the classification experiments above is less than those utilized by other methods. Moreover, epitope length could affect the epitope specificity and therefore the classification performance. Surprisingly, effect of epitope length on the classification performance has not been studied systematically in previous works. Therefore we checked the classification performance from length 7 to 15, the range in which peptides can be synthesized relatively easily (Figure 3D). We found that the F1-measure decreased from 81.37% to 73.69% and the AUC decreased from 0.88 to 0.76 with the decrease in the epitope length. However, even when training with the epitope length of 7, which is less than the epitope length utilized by BCPred and Bepipred during the training, our SVM classifier outperformed those methods. Therefore, we believe that the learning features in the best set indeed play a big role in the improved classification performance.

### Identification of the B-cell epitopes from large protein sequences

We developed a scanning procedure to identify the B-cell epitopes from large protein sequences with high confidence. The scanning procedure that we developed takes a desired peptide length, a step length and utilizes features extracted for this peptide to give it a SVM score. The scanning procedure obtained the F1-measure of 78.44% and the AUC of 0.83 with the peptide length 15, the step length of 1 and the *best feature set*. We term this predictor B-Cell epitope oracle (BEOracle).

### Validation of the BEOracle classification performance

For validating the BEOracle performance in identifying epitopes that could result in ChIP-grade antibodies, a list of 32 antibodies with known peptide epitopes was compiled from the ENCODE [2] and the modENCODE [3] project wiki pages. These antibodies have been used for identifying genome-wide binding profiles of transcription factors and DNA associated proteins by microarrays or next generation sequencing. We compared the BEOracle performance with the BCPRED and the Bepipred methods at specificity of 80%. The scanning procedure provided positive scores indicating good epitopes for 31 out of the 32 peptides and achieved an accuracy of 96.88%. The remaining 1 got the score of zero and none got the negative score that indicated poor epitopes. The BCPRED algorithm provides positive scores for 18 out of 32 (56.25%) while the BEPIPRED model provides positive scores for 17 out of 32 epitopes (53.12%; Table 1, 2).

**Table 1:** Comparision of prediction performance of BEOracle with BCPred and Bepipred on peptide antibodies used in ENCODE project

ENCODE	BEOracle	BCPred	Bepipred
BRF2-2	1	1	1

RPC155	1	0	0

BRF2-1	1	0	0

NONO	1	1	1

BDP1	1	0	0

BRF1	1	0	0

**Table 2:** Comparision of prediction performance of BEOracle with BCPred and Bepipred on peptide antibodies used in modENCODE project

modENCODE	BEOracle	BCPred	Bepipred
Rabbit anti-Stat92E	1	1	0

HDAC6-497	1	1	0

HDAC3-499	1	0	0

HDAC1-501	1	0	1

Egg (Kang)	1	1	0

chinmo 70850	1	0	1

CP190	1	0	0

CTCF-C	1	0	0

sens	1	1	1

HP1 wa191	1	1	0

CTCF-N	0	1	1

HP1 wa192	1	1	1

HDAC4a-492	1	0	1

HA	1	0	1

HDAC1-500	1	1	1

HP1c (Henikoff)	1	1	0

HDAC6-496	1	1	1

HDAC3-498	1	0	1

HDAC11-495	1	1	1

JL00012_DPY28	1	1	1

POF	1	1	1

BEAF-32	1	0	0

HDAC11-494	1	1	0

HDAC4a-493	1	0	0

anti-HTZ-1	1	1	1

HP1b (Henikoff)	1	1	1

**Figure 4:**
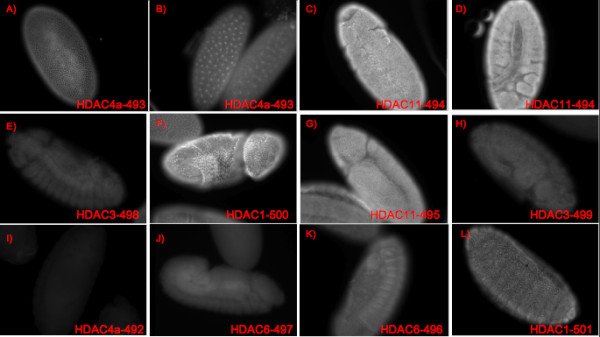
**Immunofluorescence of HDAC antibodies on Drosophila melanogaster embryos**. Experimental intensities for antibodies A-L ordered as in Table 3. A-B and C-D show images for the same antibodies.

Another validation set of 10 HDAC antibodies with known epitope sequences was also used to validate BEOracle performance. The peptides for all HDAC antibodies obtained positive BEOracle scores during the scanning procedure, thus resulting in 100% accuracy. We carried out immunofluorescence experiments for these antibodies on the *Drosophila melanogaster *embryos and annotated fluorescence intensity into four groups very good, good, low intensity and not good IF (Table 3). We sorted BEOracle scores from high to low and compared the correlation between the BEOracle score and the fluorescence intensity in a blind testing set up. There are three antibodies with "Very good" fluorescence in the top 5 versus only one in the bottom 5. Moreover, only 1 antibody in the top 5 has "low intensity" and no antibody has "not good intensity". Therefore, there is some evidence of correlation of fluorescence intensity with the BEOracle score. Although, a larger testing set is required to substantiate this claim (Table 3, Figure 4). Based on the BEOracle performance on these two independent validation sets, we can conclude that the classifier identifies B-cell epitopes from the linear protein sequences that can create antibodies with high specificity.

**Table 3:** Correlation of IF experimental results on HDAC antibody to SVM scores

Antibody_Name	SVM_Score	Comments
HDAC4a-493	0.879548	Very Good

HDAC11-494	0.767461	Very Good

HDAC3-498	0.698287	Low intensity

HDAC1-500	0.602691	Very Good

HDAC11-495	0.535022	Good

HDAC3-499	0.533935	Low intensity

HDAC4a-492	0.5196	Not good IF

HDAC6-497	0.35699	Low intensity

HDAC6-496	0.184187	Good

HDAC1-501	0.167799	Very Good

### Local information allows for the prediction of specificity in immune response for large protein regions

**Figure 5:**
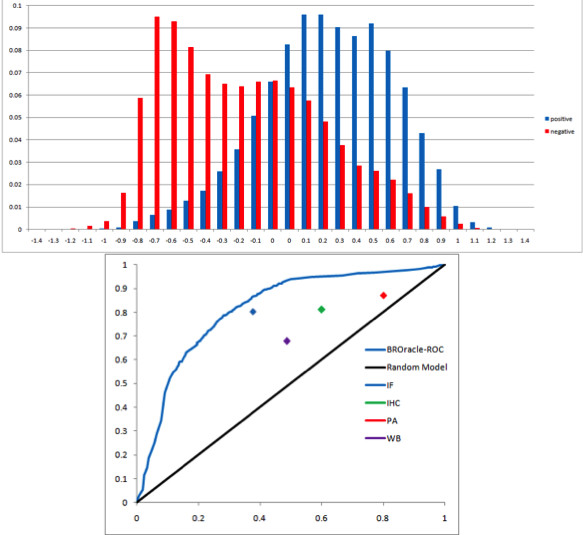
**EOracle peptide scores are predictive of the specificity in immune response for large protgein regions**. (A) BEOracle score distribution for positive and negative training examples extended to length 100. (B) Validation of BROracle classifier performance on protein atlas data: IF, IHC, PA, WB and ROC curves of BROracle on test set vs Random model.

Distributional analysis of scan scores for 4344 positive and 4344 negative training examples extended to the length 100 showed that the positive learning examples have higher frequency of positive BEOracle scores than the negative learning examples (p-value 2.2e-16; Kolmogorov Smirnov test; Figure 5A). Therefore, these extended regions containing positive and negative B-cell epitopes could be utilized to train a classifier for predicting the specificity in immune response for large protein regions or domains. However, it should be noted that the negative learning set here might contain some genuine B-cell epitopes. A second set of classifiers with different SVM kernels were trained using the BEOracle SVM scanning scores as learning features. The SVM classifier with the radial basis function kernel achieved an F1-measure of 78.45% in 5-fold cross-validation. We term this classifier the B-cell region oracle (BROracle). The BROracle classifier achieved an F1-measure of 76.00% and the ROC area of 0.8127 on the validation set (Figure 5B). The BROracle classifier has been trained such that it can handle protein regions of any length. Please see Additional File 1 for the description of the training algorithm.

### Validation of the BROracle classification performance

The classification performance of the BROracle was further validated on 600 Human protein regions of varying lengths extracted from the Human Protein Atlas database [30]. Immune response specificity information in terms of IF, WB, IHC and PA experiment results is available for these regions. The BROracle classifier achieved the highest accuracy of 76.26% for the IF and the lowest accuracy of 63.88% for the WB experiments (Figure 5B; Table 4). Therefore we can conclude that local information on antigenicity and specificity, available as BEOracle scores, can predict specificity in immune response for large protein regions. We can also conclude that specificity in immune response on large regions can be predicted without identifying discontinuous/conformational epitopes that may be present in these regions.

**Table 4:** Validation of classifier performance on Protein Atlas domains

Validation	Pos-set	Neg-set	ACC	TPR	FPR	Prec	Recall
IF	130 (Support)	60 (Non-support)	75.26%	80.29%	37.73%	80.15%	84.50%

IHC	247 (High)	77 (Very low)	71.60%	81.12%	60%	81.78%	81.12%

PA	480 (1)	80 (2)	73.93%	87.11%	80%	81.67%	87.11%

WB	258 (1~2)	159 (6~7)	63.88%	67.73%	48.93%	82.17%	67.73%

## Discussion

SVMs are widely used classifiers while performing machine learning in settings with high-dimensional feature space. They have been shown to outperform other classification methods in different learning tasks. SVMs have been used in many published studies for the B-cell epitope identification [18,21,22]. The BEOracle classifier described in this work is also an SVM classifier and provides significant improvement in the B-cell epitope prediction task, as attested on a large test set and multiple biological validation sets.

It is possible that other classification algorithms trained on the feature sets described in this work may outperform the BEOracle classifier. Instead of looking for the best classification algorithm, we have exhaustively surveyed the structural properties of proteins at the global and the residual level and identified the best sets of properties that provide improved classification performance. The novelty of this work lies in the systematic feature extraction and integration that provides information on determinants of antigenicity in protein sequences that could result on highly specific antibodies. We will compare the classification performance of different classification methods with feature sets described here in future. The BEOracle classifier requires the structural and evolutionary information from other software like BLASTP, PSIPRED, and ACCPRO20 as input. These steps are computationally intensive when performed on large data sets or on a whole proteome scale. The OLSGW scientific gateway on the TeraGrid provided the required computing power for this analysis.

Our experiments suggested that the determinants of antigenicity are largely compositional rather than positional as n-grams, secondary structure, solvent accessibility and evolutionary information were presented to the classifier largely as compositional features extracted from positive and negative learning examples. Other encodings and feature sets do not work as well. The BEOracle classifier utilizing features from only these properties provided an impressive F1-measure of 81.37% on a large test set. The classification performance improves with the increase in the peptide length as it provides more accurate estimation of epitope composition and its neighborhood. The amino acid neighborhood and evolutionary information is routinely utilized for accurate prediction of secondary structure, solvent accessibility and other structural properties. The PSIPRED method utilizes the PSSM matrices generated by the PSI-BLAST and the ACCPRO utilizes both the secondary structure predicted by the PSIPRED and the PSSM matrices generated by the PSI-BLAST. Even then features derived from these three contributed distinctly in improving the prediction performance. We saw a marked increase in the classification performance when 20-state solvent accessibility was used in the training instead of the 2-state solvent accessibility. Therefore, more granular secondary structural information may also improve the classification performance.

For any classifier, it is understandable to have some loss of prediction power due to composition of learning sets and a theoretical limit in prediction performance. For example it is estimated that secondary structure prediction possibly has an upper limit of 90% [31]. During the training of the BEOracle classifier the *best set *of learning features are selected from an exhaustive list of possible features that can be predicted. However, the learning examples were compiled from different databases with entries varying in quality and epitopes identified using different experimental methods. Moreover, the various bioinformatics methods used to predict features have their own error rates. Therefore, F1-measure of 81.37% and the AUC of 0.88 of the BEOracle classifier are indeed impressive and perhaps suggestive of the fact that our work has identified determinants of antigenicity and specificity in immune response.

This assertion is further corroborated by the identification of peptides epitopes for 32 ChIP-grade antibodies, and 10 HDAC antibodies with > 96% accuracy by the BEOracle classifier and by the performance of the BROracle classifier that successfully predict highly antigenic regions from protein sequences. The BROracle classifier only utilizes only BEOracle scores as features. It doesn't utilize any three-dimensional structure information and is unaware of any conformational epitopes that may be present in those large regions. Yet, the BROracle classifier can successfully identify those regions on a validation set with four different assay types for Human proteins extracted from the Protein Atlas database. Therefore, we believe that composition, secondary structure, solvent accessibility and evolutionary information are indeed the determinants of antigenicity and specificity in immune response as they can be used to predict antigenicity and specificity in immune response for small epitopes and for large regions.

Further improvements in the B-cell epitope prediction task can be achieved by utilizing other features. For example, knowledge of the 3-dimensional structures (3D) of proteins will provide accurate information on secondary structure and solvent accessibilities. Additional structural features like H-bonding and residue packing can be derived only from the 3D structures. Functional information like post-translational modification (e.g. known and predicted phosphorylation), protein-protein and peptide-protein interaction interfaces and Gene Ontology categories can be utilized as features. Other information on reactivity, presence of homologous sequences in the genome of antibody source/host species can also be utilized as features. Utilizing these features for predicting specificity in immune response could lead to improved performance for both continuous and perhaps discontinuous B-cell epitopes.

## Conclusions

Our results indicate that the features derived from epitope composition, secondary structure, solvent accessibility and evolutionary conservation can be used to identify linear B-cell epitopes with a very high accuracy. They also indicate that the determinants of antigenicity and specificity in immune response are largely compositional rather than positional since the learning examples for the best classifier were encoded as compositional features. The BEOracle classifier achieved an F1-measure of 81.37% and > 96% accuracy in identifying peptides for ChIP-grade antibodies for ENCODE and modENCODE projects.

The BROracle classifier predicts specificity in immune response for large protein regions. This task has not been addressed before. The BROracle classifier utilizes BEOracle scores as features and therefore predicts using the information from local features only. The BROracle classifier achieved accuracies of 75.26-63.88% on a validation set with immunofluorescence, immunohistochemistry, protein arrays and western blot results obtained from the Protein Atlas database.

Together these results suggest that immunogenicity is a local property of the protein sequences and immune response obtained from the large protein regions could be accurately predicted using local information. The knowledge about protein tertiary structure or presence of discontinuous epitopes is not required for predicting immune response obtained from large protein regions. The dataset prepared in this work and the classifier models are available for download at https://sites.google.com/site/oracleclassifiers/.

## Methods

### Non-redundant Dataset

We have prepared a large dataset containing positive and negative training examples by combining entries from immune epitope database (IEDB), BCIPEP and AntiJen databases. We parsed these databases and matched equivalent entry fields (Additional File 1, Table S1). We extended these epitope sequences equally on both the sides to get epitopes of a final length of 100 amino acids using the Uniprot identifiers associated with them. The redundant peptides were removed from this set as the presence of redundant examples in the learning set could provide misleading results. The final dataset contain 6581 positive and 9353 negative epitope examples. The extended sequences allowed for an accurate prediction of structural properties for the epitope sequences as well as getting uniform length peptides.

### Open Life Science Gateway for predicting protein structural properties

We have developed and utilized the Open Life Science Gateway (OLSGW; Wu *et al*, under review) for predicting the structural properties of protein sequences. OLSGW is a science gateway designed to rapidly deploy and offer most commonly used bioinformatics software and community databases, as web based services in a standalone or a workflow based formats on the front end while interacting with the TeraGrid resources through reliable middleware for computing. OLSGW makes a vast amount of computing resources at the Argonne National Laboratory and 11 other partner sites freely available for solving complex biological problems without exposing users to the complexity of Grid computing. OLSGW webportal is available at (http://gw25.quarry.iu.teragrid.org:8080/gridsphere/gridsphere).

The current version of the OLSGW offers software for sensitive sequence database searches (PSI-BLAST [32] and HMMER [33]), multiple sequence alignments (CLUSTALW [34] and MUSCLE [35]), secondary structure prediction (PSIPRED [36]), solvent accessibility prediction (ACCPRO [37]), disorder prediction (VSL2 [38]), 3-dimensional homology modeling (MODELLER [39]), and identification of a variety of protein signatures (Interpro [40]) including sequence and structural domains, signal peptide, transmembrane regions and low-complexity regions. After a quick and automated registration process, the OLSGW web portal allows submission of a single or multiple query protein sequences in FASTA format. OLSGW searches for available computing nodes on the TeraGrid partner sites, submits the job on an appropriate queuing system, monitors them in real time for completion and resubmits failed jobs. We utilized the OLSGW to generate BLASTP PSSM, secondary structure, solvent accessibility, disorder and low complexity information for more than 18,000 sequences that belong to the training and the validation sets. These calculation required more than 1700 hours or 28 days woth of CPU time. It was finished under 24 hours by the means of the TeraGrid resources that was facilitated by the OLSGW.

### Feature Calculation and Extraction

An exhaustive prediction of structural properties was carried out using well-known bioinformatics software. We extracted features providing evolutionary, structural and compositional information to the classifier. See Additional File 1 for examples of possible feature space for proteins and different ways of encoding them. Here we describe encoding for only those features that were worth reporting in the work described here. For each learning example PSI-BLAST [41] was run for 3 iterations and a p-value cutoff of 1e-03 on *nrfilt *(filtered *non redundant protein *sequence database from the NCBI) database to generate the PSSM that provides evolutionary conservation information for each position of the query sequence. The *nrfilt *database is available for download with the PSIPRED package [36]. For each amino acid the three-state secondary structure was predicted with the PSIPRED package associating probability of being in helix, beta-strand or coil to them. The twenty-state solvent accessibility was predicted with ACCpro20 [37] providing probability of being accessible to each amino acid. The two state disorder was predicted with the VSL2 algorithm [38] assigning probability of being in disordered state to each amino acid. The low complexity was predicted with the *seg *algorithm [42]. The compositional information (e.g. percentage of amino acids in helix) was calculated for each structural feature for the original (non-extended) epitopes.

We utilized a total of 53633 learning features combining different types of information. N-varying from 1 to 5 provided the SVM with 20, 230, 1770, 10625 and 53129 features with the increasing number of n. They provide the SVM with compositional information. For each epitope we utilized evolutionary conservation information from the PSI-BLAST generated PSSM file columns 21 to 40. Secondary structure contributes with 20*3+3 features. The first 20 features provide the average of 3-state prediction scores for each amino acid present in the peptide. The last 3 features provide composition of the peptide in terms of 3 assigned states from PSIPRED output. 20-state solvent accessibility and disorder contributes 20 features each in the way similar to the secondary structure. The low complexity prediction contributes one feature for the whole epitope. The learning features were scaled using *svm-scale *function in the libsvm [43] to make them comparable to each other and provided as input to the SVM classifier. The feature extraction procedure for the BROracle classifier is described in Additional File 1.

### Classification Experiments and comparison with other methods

We have carried out SVM classification experiment for 5 different lengths 7, 9, 11, 13, and 15, and 5 different kernel functions including linear, polynomial kernels of degrees 2 and 3, radial basis function (RBF), and sigmoid for each featureset. The classification parameters like F1-measure, precision, recall and accuracy reported for the training set are sum of the 5-fold cross validation. Each partition of our dataset had 748 positive and negative learning as well as test examples. The SVM^light ^package [44] implementing support vector machines with fast optimization algorithm and multiple kernel functions was used for classification experiments. During the learning phase the SVM algorithm requires labeled positive and negative learning examples as features. Once a model is trained, test examples are read and a score is given to each example. In this work, test examples with scores greater than zero are considered peptides that could be good B-cell epitopes and examples with scores less than zero are considered peptides that wouldn't be good B-cell Epitopes. We compared classification performance of our best classifier with BCEPRED that is based on propensity scales, BCPred that utilizes an SVM based classifier [21] and Bepipred [1] that utilizes a hidden markov model. The BCEPRED method implements a total of seven classical propensity scales that utilize protein physicochemical properties like hydrophilicity [5], flexibility [45], accessibility [7], turns [46], antigenicity [11], and polarity [8,9]. More information can be found on http://www.imtech.res.in/raghava/bcepred/bcepred_algorithm.html.

### Datasets for Biological Validation

We validated the BEOracle classifier performance in three different ways: 1) on a large test set, 2) using a list of peptides that were used in generating antibodies for carry out chromatin immunoprecipitation experiments as a part of ENCODE and modENCODE projects and 3) a set of HDAC antibodies for which Immunofluorescence data was generated on *Drosophila melanogaster *embryos as a part of the modENCODE project.

For the validation of the BROracle classifier, a large test set, and 600 protein regions extracted from the Protein Atlas database are available [30]. These 600 protein regions have assay quality scores for immunofluorescene (IF; support vs. non-support), immunohistochemistry (IHC; High vs. very low), western blot (WB; 1-2 vs. 6-7) and protein array (PA; 1 vs 2).

## Abbreviations

BEOracle: B-Cell Epitope Oracle; SVM: support vector machine; BROracle: B-Cell Region Oracle; ENCODE: Encyclopedia of DNA elements; modENCODE: model organism of Encyclopedia of DNA elements; HMM: hidden markov model; OLSGW: open life science gateway; RBF: radial basis function kernel; AUC: area under curve; IF: immunofluorescence; WB: western blot; IHC: immunohistochemistry; PA: protein array.

## Competing interests

The authors declare that they have no competing interests.

## Authors' contributions

YW carried out the SVM classification experiments for various feature combinations and other analysis. WW ran the OLSGW analysis for calculating structural features. NN carried out the immunofluorescence experiments on Drosophila embryos. KPW and CL provided conception, funding and critical inputs. PKS prepared the datasets, designed and carried out SVM classification experiments, interpreted results and wrote the manuscript. All the authors read and approved the manuscript.

## Supplementary Material

Additional file 1 **supporting material to the main manuscript**. contains supplementary text, figures and tables Click here for file
