# Supplementary material for: Determinants of antigenicity and specificity in immune response for protein sequences
Source: BMC Bioinformatics. 2011 Jun 21;12:251. doi: 10.1186/1471-2105-12-251 (PMC3133554; doi:10.1186/1471-2105-12-251)
Supplement: Additional file 1 — supporting material to the main manuscript. contains supplementary text, figures and tables [file 1471-2105-12-251-S1.DOC]

**Title: Determinants of Antigenicity and Specificity in Linear B-cell Epitope Prediction in Protein Sequences**

**Authors**:

Yulong Wang, Wenjun Wu, Nicolas N Negre, Kevin P White, Cheng Li and Parantu K Shah*

**Supplementary Material**

**Index**

1. **Glossary of Important Terms**
2. **Distribution of Epitope lengths in learning set**
3. **SVM learning features**
4. **Examples of feature encoding for support vector machines.**
5. **Definitions of precision, recall and F1-measure**
6. **SVM learning performance with physico chemical scales with various encodings**
7. **Derivation of features for BROracle**
8. **Comparison with BCPred on El-manzalawy dataset and importance of negative learning examples**

**1) Glossary of Terms**:

1. Epitope: An epitope is a chemical structure recognized by specific receptor of the immune system (e.g. antibodies, MHC or T-cell receptors)
2. Antigen: An antigen is any substance that can be recognized by an antibody (or TCR)
3. Immunogen: An immunogen is an antigen that can activate an immune response. (some antigens e.g. haptens can’t activate immune response on their own).
4. Continuous Epitope: epitopes that occur in a sequential fragment from a protein sequence (also called linear or sequential epitopes).
5. Discontinuous Epitope: epitopes that is composed of several fragments scattered along the protein sequence (also called conformational epitopes)
6. Immunodominant: The peptide which result in 2-3 fold increase in anti-peptide antibodies in comparision to reference or control peptide and produce protective immunity.
7. Immunogenicity: The peptide which result in 1 fold increase in anti-peptide antibodies in comparision to reference or control peptide and produce immunity.
8. Null Immunogenicity: The peptide that results in base level of antibody production as produced by control peptide.

**2) Distribution of epitope length in the learning sets**

We parsed IEDB , BCIPEP and AntiJen databases and matched equivalent entry fields (S Table 1). In the learning dataset derived by merging, positive and negative examples are of various lengths (S. Figure 2). It is possible that longer epitopes possess more information than shorter ones. Moreover, it was one of the aims of our work to assess length dependence in the classification performance. To get uniform learning sets, we extended the lengths of epitopes by deduction or addition to uniform lengths.

| **BCIPEP** | **Antijen(linear)** | **IEDB(linear)** |
| --- | --- | --- |
| **Sequence** | Epitope | Linear_sequence |
| **Model_Studied** | Ab Source | Immunized_species |
| **Antibody** | Antibody | Antibody_Type |
| **-** | Ab nomenclature | Heavy_Chain_Isotype |
| **DbReference** | SwissProt Ref | Source |
| **Immunogenicity** | Reactivity | Measurement_Char_Value |
| **Neutralization** | - | - |
| **Source** | Peptide Source | Epitope_Source |

**Supplementary Table 1:** Context information and field comparison


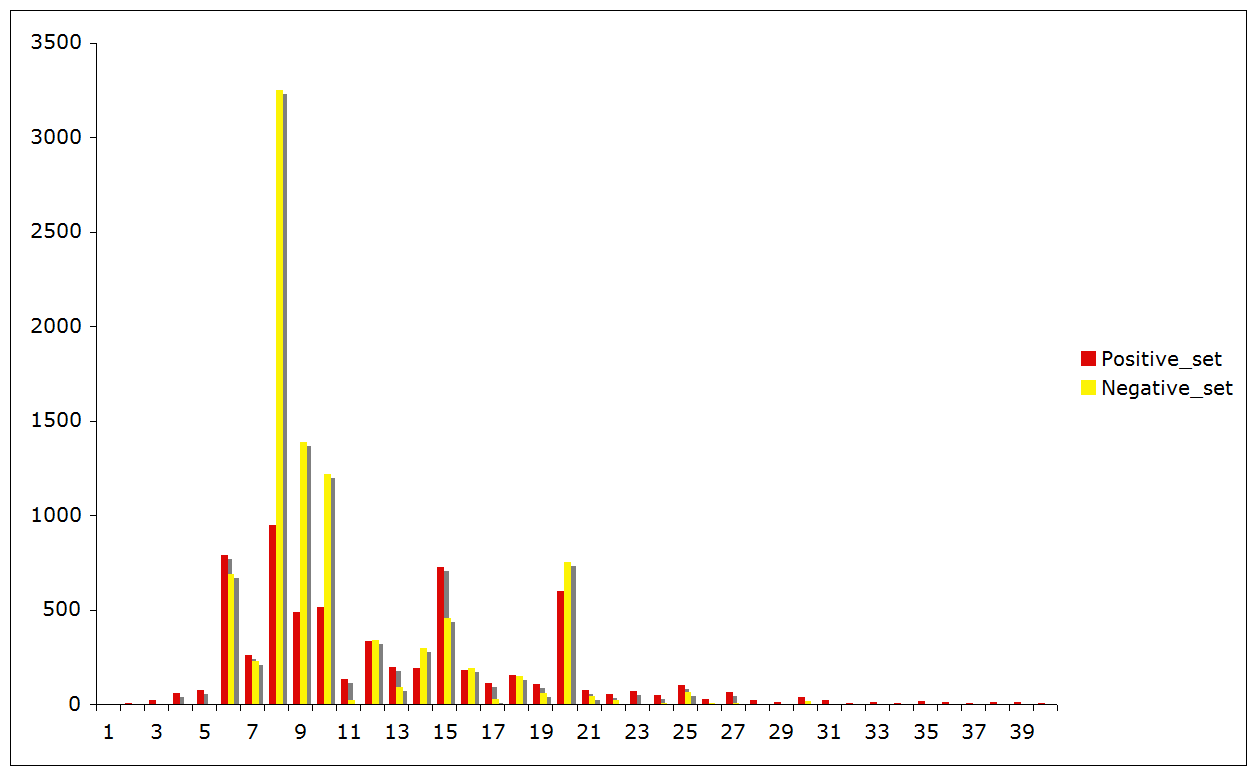


**Supplementary Figure 2:** Epitope length distribution for positive and negative learning examples

**3) SVM learning features**

**Supplementary Figure 3**: A partial list of learning features that could be given as input to SVMs. These features can be divided into two broad classes. Features that can be derived from overall protein sequences or aggregate features and features that could be properties of individual amino acids or residues features. A lot of residues features can have accurate values when 3D structures is known by the means of X-ray crystallography or NMR. Some of them could also be predicted using Bioinformatics algorithms. Features in red are considered in this study.

**4) Examples of feature encoding for support vector machines.**

**Supplementary Figure 4**: Feature Encoding Examples for providing amino acid sequences to support vector machines. For example, to encode 1-gram feature each amino acid is given one feature number and frequency as the value. For encoding bi-gram features each amino acid pairs is given a feature number. Bottom panel shows how to derive n-grams for n= 1, 2 and 3 for an example peptide. N-grams that are permutations of the same amino acids are considered equivalent.

**Supplementary Figure 5**: Positionwise and windows based encoding for providing amino acid sequences to support vector machines. For position wise encoding each position is given 20 features corresponding to 20 amino acids. Note different feature numbers for amino acid Q in the upper panel for the example peptide of length 8. In window based encoding as shown in the bottom panel, a window is chosen (e.g. 7) the central amino acid is given a score that is sum of its neighboring (e.g. +/- 3) amino acids using a physico-chemical scale as done in “classical” methods for B-cell epitope prediction. It is analogous to “classical” methods for B-cell epitope prediction.

**5) Definitions of Precision, Recall and F1-measure**

The precision and recall of classifier are defined as follows:

**
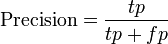
 and
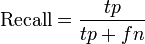
**

where TP, TN, FP and FN are true positives, true negatives, false positives and false negatives. The F1-measure is defined as below:


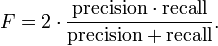


**6) SVM learning performance with physico-chemical scales with various encodings**

**6.1 Learning performance with n-gram features normalized using background frequency of amino acids.**


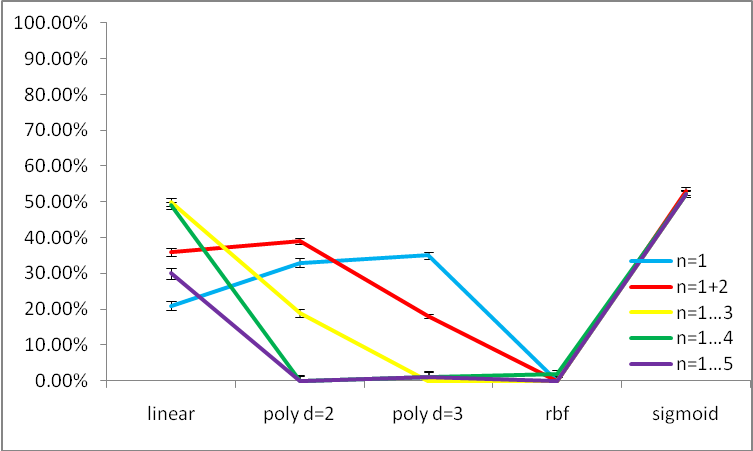
**Supplementary Figure 5:** Learning perfornace of features normalized using background frequency of amino acids from Uniprot database. SVM Learning performance as defined by F1-measure (Y-axis) degrades when the features are normalized using background amino acids frequencies calculated using Uniprot database. For example, n= 1..5 un normalized features provides F1-measure of 73.95 % and normalized features provides F1-measure of 0 % in 5-fold cross validation.

**6.2 Windows and position based encoding with different physico chemical scales doesn’t perform well**

**6A**

**6B**


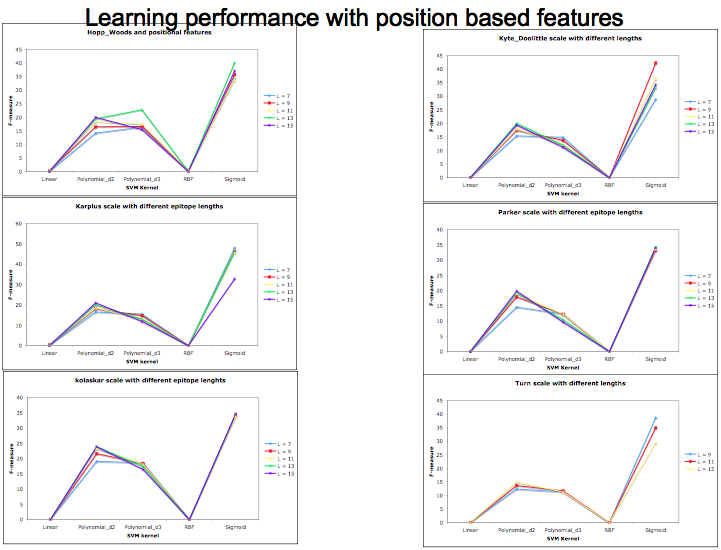


**Supplementary Figure 6**: SVM performance on various physico-chemical scales. Hopp-woods scale for Hydropathicity , Karplus scale for protein flexibility, Kyte-Doolittle scale for hydrophobicity , Kolaskar-Tongaonkar scale for antigenicity , Parker scale for hydrophilicity and Pellequer turn scale that provides propensity value for each aminoacid is used for SVM learning. (A) SVM learning performance as F1-measure (Y-axis) with a total of 20 aminoacid composition features with each amino acid assigned the value using one of propensity scales. X-axis shows the SVM kernel. The sigmoid kernel doesn’t provide a model in this experiments (B) SVM classification performance on epitopes of different lengths for various physico-chemical scales. Hopp-woods, Karplus and Kolaskar on the left panel and Kyte-Doolittle, Parker and Turn scales on the right panel. None of the SVM model provide satisfactory learning performance in terms of F1-measure. Learning performance using different window sizes produce similar results.

**7) Derivation of features for BROracle**

The B-cell region oracle classifier that could predict the specificity in immune response for antibodies generated against large protein regions. It takes the BEOracle scores as input features. The classifier was trained on positive and negative examples of protein regions of length 100. The regions of length 100 were scanned with the length 15 and step size of 1 to generate the input features for BROracle. For each protein sequence in our training set, sorted top 60 scores out of 86 BEOracle scan scores of were utilized as training features.

The validation sequences in Human extracted from ProteinAtlas database range from 70 to 1500. We generate 60 features (F) for sequences greater or less than length (X) 100 amino acids and S be the sorted scores.

Set length = X, feature = {F(1),F(2),…,F(60)}, sorted scores = {S(1),S(2),…,S(X-14)} and S(1)>S(2)>…>S(X-14).

Consider two situations:

Situation 1: X>100,

For 0<n<61,

F(n)=S([(X-14)*(1/86)*n]) (Note that function [x] is the largest integer which is no larger than x)

Situation 2: X<100,

For 0<n<61,

F(n)=S([(X-14)*(1/86)*n]) (Note that function [x] is the largest integer which is no larger than x)

If F(n)=F(n+1), then F(n+1)=0.

**8) Comparison with BCPred on El-manzalawy dataset and importance of negative learning examples**

| **Classifier** | **Accuracy** | **Precision** | **Recall** | **F1-measure** | **AUC** |
| --- | --- | --- | --- | --- | --- |
| **BEOracle negative 12** | 0.7255 | 0.7930 | 0.7282 | 0.7592 | 0.7951 |
| **BEOracle**  **negative 14** | 0.7301 | 0.8013 | 0.7249 | 0.7612 | 0.8019 |
| **BEOracle**  **random 12** | 0.7145 | 0.7730 | 0.7082 | 0.7390 | 0.7813 |
| **BEOracle**  **random 14** | 0.7200 | 0.7823 | 0.7109 | 0.7449 | 0.7881 |
| **BCPred 12** |  |  |  |  | 0.7135 |
| **BCPred 14** |  |  |  |  | 0.7145 |

Comparison of the BEOracle classification performance at lengths 12 and 14 when utilizing random sequences as negative examples from the El-Manzalawy dataset versus real negative training examples. The positive training examples are from the El-Manzalawy dataset in each case.

**References**:

1. Vita R, Z.L., Greenbaum JA, Emami H, Hoof I, Salimi N, Damle R, Sette A, Peters B, *The immune epitope database 2.0.* Nucleic Acids Res, 2010.

2. Saha, S., Bhasin,M and Raghava,G.P.S, *Bcipep: A database of B-cell epitops.* BMC Genomics, 2005.

3. Saha, S., Bhasin,M and Raghava,G.P.S, *Bcipep:A database of B-cell epitopes.* Nucleic Acids Research, 2005.

4. McSparron H, B.M., Zygouri C, Doytchinova IA, Flower DR, *JenPep: A Novel Computational Information Resource for Immunobiology and Vaccinology.* J Chem Inf Comput Sci, 2003.

5. Blythe MJ, D.I., Flower DR, *JenPep: a database of quantitative functional peptide data for immunology.* Bioinformatics, 2002.

6. Kyte J., D.R.F., *Amino acid scale: Hydropathicity.* J Mol Biol, 1982.

7. G.E.Schulz, P.A.K.a., *Flexibility scale.* Naturwissenschaften, 1985.

8. Doolittle, J.K.R.F., *A simple method for displaying the hydropathic character of a protein.* J Mol Biol, 1982.

9. P.C.TongaonKar, A.S.K.a., *Antegenic propensity scale.* FEBS, 1990.

10. J.M.D. Parker, D.G., and R.S. Hodges, *Hydrophilicity scale.* Biochemistry, 1986.

11. J-L Pellequer, E.W., Regenmortel MHV, *Turns scale.* Immunology Letter, 1980.

12. Uhlen, M.e.a., *A human protein atlas for normal and cancer tissues based antibody proteomics.* Mol. Cell. Proteomics, 2005.
